# QSPR designer – employ your own descriptors in the automated QSAR modeling process

**DOI:** 10.1186/1758-2946-4-S1-P37

**Published:** 2012-05-01

**Authors:** Ondřej Skřehota, Radka Svobodová Vařeková, Stanislav Geidl, Michal Kudera, David Sehnal, Crina-Maria Ionescu, Jan Žídek, Jaroslav Koča

**Affiliations:** 1 NCBR and CEITEC - Central European Institute of Technology, Masaryk University, Brno, CZ 625 00, Czech Republic; 2 BioPeta, Brno, CZ 625 00, Czech Republic; 3 CEITEC, Brno University of Technology, Brno, CZ 616 00, Czech Republic

## 

The prediction of physical and chemical properties of molecules is a very important step in the drug discovery process. QSAR and QSPR models are strong tools for predicting these properties. The models employ descriptors and statistical approaches to provide an estimation of the desired property. An abundance of descriptors and QSAR/QSPR models were published, but the prediction of some properties (i.e., pK_a_, logP) is still a challenge [1]. For this reason, researchers are perpetually working on identifying new descriptors and analyzing their performance in newly designed models. The process of design, parameterization and evaluation of a QSAR/QSPR model is relatively complicated. Therefore, several software tools for its automation are currently under development [2,3]. These tools are very useful if we wish to apply some of many descriptors which they implement. But if we need to use other descriptors or test our own, we require a different solution.

We introduce a new version of the software QSPR Designer that fulfils the above mentioned requirement. Specifically, the user can easily and quickly add his own module for descriptor calculation into QSPR Designer. Alternatively, descriptors can also be obtained from an input file. When the descriptors are available, QSPR Designer allows the user to include them to the model, to validate this model and to perform further tasks. The functionality of QSPR Designer is demonstrated on three case studies, in which the user gradually tests and improves his ideas of how to predict pK_a_. The first case study is focused on the question whether pK_a_ has any relation with atomic charges. The second case study tests if we can use charges and a simple similarity-based model to predict pK_a_. The third case study analyzes several QSPR models predicting pK_a_ from charges.
